# Causes for Hospitalizations at Upazila Health Complexes in Bangladesh

**DOI:** 10.3329/jhpn.v28i4.6047

**Published:** 2010-08

**Authors:** Sirajuddin Ahmed, A.K. Siddique, Anwarul Iqbal, F.K.M. Nurur Rahman, Md. Noor Islam, Md. Arif Sobhan, Md. Rafiqul Islam, R.B. Sack

**Affiliations:** ^1^ Public Health Sciences Division, ICDDR,B, GPO Box 128, Dhaka 1000, Bangladesh; ^2^ Department of Public Health, King Fahd General Hospital, PO Box 1726, Tabuk, Saudi Arabia; ^3^ BRAC International Liberia, Allision Road, Congo Town, Monrova, PO Box 2549, Liberia; ^4^ School of Medicine and Public Health, David Maddisson Building, University of Newcastle, NSW 2300, Australia; ^5^ Department of International Health, Johns Hopkins Bloomberg School of Public Health, 615 North Wolfe Street, Baltimore, MD 21205, USA

**Keywords:** Hospitalization, Morbidity, Rural health services, Bangladesh

## Abstract

Morbidity and mortality data are important for planning and implementing healthcare strategies of a country. To understand the major causes for hospitalizations in rural Bangladesh, demographic and clinical data were collected from the hospital-records of five government-run rural health facilities (upazila health complexes) situated at different geographical regions of the country from January 1997 to December 2001. During this period, 75,598 hospital admissions in total were recorded, of which 54% were for male, and 46% were for female. Of all the admissions, diarrhoeal disease was the leading cause for hospitalization (25.1%), followed by injuries (17.7%), respiratory tract diseases (12.6%), diseases of the gastrointestinal tract (10.5%), obstetric and gynaecological causes (8.5%), and febrile illnesses (6.7%). A considerable proportion (8.3%) of the hospitalized patients remained undiagnosed. Despite the limitations of hospital-based data, this paper gives a reasonable insight of the important causes for hospitalizations in upazila health complexes that may guide the policy-makers in strengthening and prioritizing the healthcare needs at the upazila level in Bangladesh.

## INTRODUCTION

The health status of people of a nation is reflected by their morbidity and mortality patterns. The information is important for planning and implementing healthcare strategies and for monitoring healthcare services of the country (1–3). In many developing countries, accurate population-based morbidity data are largely deficient or absent (4). This may also be true for Bangladesh. The country is inhabited by nearly 125 million people (5) and is divided into 507 administrative units called upazila (subdistrict), with an average population of ∼200,000 in each subdistrict (6). More than 80% of the people of Bangladesh live in rural areas (5).

The Government provides healthcare services to its rural people through health facilities called upazila health complex (UHC) at the upazila level and through union subcentres at the union level (smallest administrative unit). Many non-governmental organizations (NGOs) also provide healthcare services through community clinics and similar other establishments (7,8). In addition, the informal sectors provide healthcare services at the village level (9). In the government healthcare-delivery systems at the upazila level, there are 460 UHCs—each with 31 beds—to provide inpatient care to its population (8). It also provides outpatient care, primary healthcare, family-planning services, and other preventive healthcare services to its population (8,10). Each UHC provides healthcare services to a population of about 100,000 to 400,000 depending on its size (8). In terms of service-delivery, the UHCs represent 31% of the government health sector, signifying their importance as a major contributor to healthcare service in Bangladesh (8). The UHCs are also the referral centres for a number of grassroots-level community clinics (10). Medical graduates, along with the paramedics and nurses supported by personnel for laboratory services and supplies, are responsible for healthcare services provided by the UHC (10).

Each UHC follows a disease-reporting system as recommended by the Ministry of Health and Family Welfare, Government of Bangladesh. In the absence of adequate diagnostic facilities, clinical evaluations of patients conducted by the attending physicians are the mainstay of diagnosis of illness. However, information collected by the system is not very often analyzed for useful purpose. Therefore, very little information is available on the major causes for hospitalizations at the UHCs in Bangladesh for the policy-makers to prioritize the healthcare needs at the upazila level.

In most communications, we have morbidity data that are derived either by a community survey or by a survey on outdoor patients attending the health facilities. In this paper, we have attempted to understand the major causes for hospitalizations in different age-groups and in both male and female in rural health facilities to guide the policy-makers in strengthening and prioritizing healthcare needs at the upazila level that would benefit the rural community in Bangladesh.

## MATERIALS AND METHODS

**Fig. 1.**
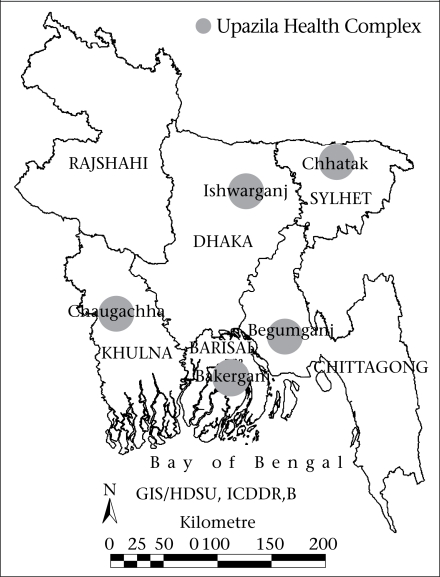
Locations of upazila health complexes

During January 1997–December 2001, while conducting cholera surveillance at five UHCs, surveillance physicians from the Epidemic Control Preparedness Unit of ICDDR,B (International Centre for Diarrhoeal Disease Research, Bangladesh) collected data from hospital-registers on all hospitalized patients. The different locations of the upazilas (subdistricts) are shown in Figure 1. Data collected included demographic information and clinical diagnosis made by the attending physician. Data collected were later entered into a microcomputer and validated by double entry and logical checks. The compiled data of categorized causes for hospitalizations presented in this study are essentially similar to those of the disease-reporting system available at the UHC. We followed the age-group classifications that were in use at the UHCs for reporting different illnesses to the district level during the above time period. The differences in the proportion of different causes for hospitalizations between two groups were done by the chi-square test.

## RESULTS

In total, 75,598 hospital admissions were recorded in five UHCs during the five-year period (1997–2001). The annual hospitalization rate was 7.7 per 1,000 people (range 4-14 per 1,000 people per year). Of the total number of hospitalizations, 54% were male, and 46% were female. More than one-third of the admissions were from the union where the health facility is situated. This observation was consistent for all the five UHCs.

The causes for hospital admissions in broad categories are shown in Table 1. Of all the causes, diarrhoeal disease was the leading cause for hospitalization (25.1%), followed by injuries (17.7%), respiratory tract diseases (12.6%), diseases of the gastrointestinal tract (10.5%), obstetric and gynaecological causes (8.5%), and febrile illnesses (6.7%). Over 8% of the hospitalized patients, however, remained undiagnosed. Since obstetric and gynaecological causes are strictly limited to female hospitalizations, we restricted our subsequent analysis for the remaining five major causes that together accounted for 72.6% of the total number of hospitalizations. These were further analyzed to see the distribution of their age and sex.

**Table 1.** Distribution of hospitalized patients by type of illness in 5 rural hospitals of Bangladesh, 1997-2001

Type of disease	No. of cases (n=75,598)	%
Diarrhoeal diseases	18,999	25.1
Injury	13,369	17.7
Diseases of the respiratory tract	9,549	12.6
Diseases of the gastrointestinal tract	7,911	10.5
Obstetric and gynaecological causes	64,31	8.5
Undiagnosed	6,282	8.3
Febrile illnesses	5,076	6.7
Surgical causes	1,397	1.8
Diseases of the cardiovascular system	1,374	1.8
Diseases of the renal system	1,245	1.6
Diseases of the nervous system	1,171	1.5
Communicable diseases	751	1.0
Intestinal parasitic diseases	641	0.8
Nutritional deficiency	530	0.7
Metabolic diseases	383	0.5
Others	489	0.6

As injuries are rapidly becoming the leading cause of morbidity among the people not only in developed countries but also in developing countries, we analyzed the injury cases (Table 2) and found that assaults of all types accounted for the majority (69%) of the admissions from injuries while 9% of the admissions due to injuries were due to road traffic accidents. A number of other injuries, such as suicides, poisoning, drowning, domestic falls, animal and insect bites were responsible for 22% of the admission due to injuries.

**Table 2.** Distribution of hospitalized injury cases in 5 rural hospitals of Bangladesh, 1997-2001

Type of injury	No. (n=13,369)	%
Assault	9,221	69.0
Road traffic accident	120	9.0
Others	2,948	22.0

Table 3 shows the distribution of patients hospitalized due to the five leading causes in different age-groups. In children aged less than five years, diarrhoeal (50.9%) and respiratory diseases (41.5%) accounted for 92.4% of all the leading causes for admissions in this age-group. Among the older children (5-14 years), diarrhoeal diseases alone accounted for almost 50% of the hospitalizations. The remaining cases were due to other four causes (Table 3). In contrast, admissions due to injuries in the age-group of 15-45 years accounted for 41.4% of the admissions while diarrhoeal diseases and diseases of the gastrointestinal tract each accounted for slightly over 22% of the admissions in this age-group. Febrile illnesses accounted for 10.4% of all the admissions in this age-group. In the age-group of over 45 years, 31.7% of all the admissions were due to injuries while diarrhoeal diseases and diseases of the gastrointestinal tract accounted for 22.7% and 22% of the admissions respectively. Admissions due to respiratory diseases and febrile illnesses accounted for 12.8% and 10.8% respectively in this age-group.

**Table 3.** Percent distribution of hospitalized patients by age-group and 5 leading causes of illness in 5 rural hospitals of Bangladesh, 1997-2001

Type of disease	<5 years (n=17,815)	5-14 years (n=6,814)	15-45 years (n=23,746)	>45 years (n=6,529)
Diarrhoeal diseases	50.9	49.9	22.4	22.7
Watery diarrhoea	49.9	47.9	21.2	21.5
Dysentery	1.0	1.9	1.2	1.2
Injuries	2.3	16.6	41.4	31.7
Assault	0.5	5.8	29.8	26.8
Road traffic accident	0.2	3.0	3.7	3.5
Others	1.6	7.8	7.9	1.4
Respiratory diseases	41.5	6.9	3.7	12.8
Pneumonia	18.0	1.7	0.3	0.7
Acute respiratory infection	22.3	2.6	0.3	0.3
Tuberculosis	0.02	0.03	0.2	0.2
Chronic obstructive pulmonary diseases	0.7	1.3	1.5	6.7
Respiratory tract infection	0.5	1.0	1.3	3.1
Others	0.1	0.3	0.2	1.8
Gastrointestinal tract diseases	1.9	14.2	22.1	22.0
Peptic ulcer diseases	0.1	0.6	5.0	5.0
Abdominal pain	1.8	13.4	16.6	18.1
Others	0.1	0.2	0.4	0.5
Febrile illnesses	3.4	12.4	10.4	10.8
Fever without definite diagnosis	3.2	10.6	8.5	8.8
Enteric fever	0.1	1.6	1.7	1.2
Malaria	0.03	0.13	0.15	0.2

**Fig. 2.**
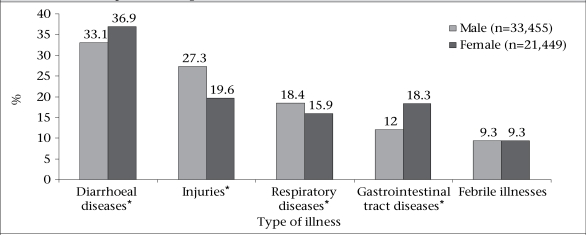
Percent distribution of hospitalized patients by gender and 5 leading causes of illness in 5 rural hospitals of Bangladesh,1997-2001

The distribution of the leading causes for hospitalizations by gender is shown in Figure 2. The proportion of hospitalizations due to injuries and respiratory diseases was significantly higher (p<0.001) among males than among females. In contrast, the proportion of hospitalizations due to diarrhoea and other gastrointestinal diseases was significantly higher (p<0.001) among females. There was no significant difference in the proportion of hospitalizations due to febrile illnesses between males and females.

*p<0.001

## DISCUSSION

The findings showed that diarrhoeal diseases continue to be the leading cause for hospitalizations in rural areas; watery diarrhoea and acute respiratory infection (ARI) were the two major causes for hospitalizations in children aged less than five years; injuries requiring hospitalizations have become a major public-health concern, especially among the adult rural population (aged 15 years and above); and a high proportion of hospitalized patients remains undiagnosed.

In recent times, there has been a considerable decline in mortality due to diarrhoeal diseases in Bangladesh (8,11–12). However, morbidity due to the disease remains still high (13) that leads to the increased need for hospitalization compared to other diseases as has been revealed through this study. The delivery of message, particularly in the rural community, that diarrhoea is a life-threatening disease and, in severe form, may quickly cause deaths if not treated may explain the higher proportion of hospitalizations due to the disease.

Several factors, such as improvement in primary healthcare services, high coverage of Expanded Programme on Immunization (EPI), widespread use of oral rehydration solution, and improvement in water and sanitation conditions, have contributed to a marked reduction in deaths of infants and children due to two major causes—diarrhoea and respiratory infection (14). This information led us to think that morbidity due to the above two diseases has also decreased. In contrast, we have observed that more than 90% of the admissions among children aged less than five years were due to watery diarrhoea and ARI, reflecting the fact that they still remain as major causes of childhood morbidity, particularly in rural areas. This observation is consistent with the findings documented in a report of the World Health Organization (13).

Morbidity, mortality, disabilities, and socioeconomic burden due to injuries, such as road traffic accidents, burns, poisoning, suicides, and assaults, have become the major public-health issues in countries of the South-East Asia region (13). In this paper, injuries were the second most common cause for hospitalizations, indicating that this health problem is rapidly becoming the leading cause of morbidity among the people not only in developed countries but also in developing countries. The rate of hospitalization (17.7%) due to injuries of all types during the period is consistent with the findings of a hospital-based study in Bangladesh where 20% of all admissions were due to injuries (15).

The reason for such a high rate of hospitalization in rural areas due to injuries is not clearly understood. We have shown that assaults were the leading cause (69%) for hospitalization due to injuries. It is known that, in rural areas of Bangladesh, the assault cases are largely associated with dispute over ownership and demarcations of land. This implication may have influenced the observed proportion of hospitalization due to injuries. However, this notion could only be clarified by a further study.

A considerable proportion (8.3%) of the admissions remained undiagnosed. This could probably be due to the lack of having adequate laboratory facilities at the rural health centres (16). Hospitalizations due to surgical causes, cardiovascular diseases, and diseases of the renal system and nervous system were only 6.7% of the total number of admissions, and again, this could be linked to inadequate diagnostic and treatment facilities at the health centres. This inadequacy at the health facilities could also be responsible for the lower rate of hospitalizations due to diseases, such as tuberculosis, enteric fever, malaria, and nutrient deficiency disorders that are common in Bangladesh.

The annual hospitalization rate in the healthcare facilities was 7.7 per 1,000 people, and more than one-third of the hospitalizations were from the area where the health facilities are situated. Since we do not have the information to explain this low annual hospitalization rate, factors, such as distance of the health facilities from the place of living and the expense associated, along with the cost of healthcare services (17–20), could be responsible for this. The shortage of essential drugs and unavailability of adequate diagnostic procedures could also be responsible for such a low hospitalization rate (16). Inequality in access to healthcare services between the rich and the poor may also explain this situation (8).

Results of studies in different countries have demonstrated gender inequalities in accessing to healthcare services (12,21–22). In Bangladesh, women are less privileged in terms of access to healthcare services than men, especially when they are subjected to violence leading to physical injuries (8). However, the reasons for the observed significant difference in hospital admissions due to the five major causes between males and females were not explored

### Limitations

Hospital-based data have some limitations that are more commonly encountered in resource-poor settings, and all these could have affected our study findings. In most cases, the healthcare services provided by the Government in rural areas are being underused for various reasons. Often there is lack of adequate information, and these are of poor quality. Further, lack of adequate diagnostic tools at the upazila health facilities might be responsible for missing more specific diagnosis of the diseases.

### Conclusions

Despite the limitations, this paper gives an insight of the important causes for hospitalizations in the UHCs. In course of time when there is widespread use of microcomputers, the individual UHCs will be able to analyze their data on admitted patients in a more systematic way to aid in the planning and evaluation of their services. However, the information provided here is expected to help the policy-makers take necessary measures in strengthening and prioritizing the healthcare needs at the upazila health facilities in Bangladesh.

## ACKNOWLEDGEMENTS

The study was partially funded by the National Institutes of Health (Grant No. IROI A139129-01A1). ICDDR,B is supported by donor countries and agencies, which provide unrestricted support for its operation and research. Current donors providing unrestricted support include the Australian Agency for International Development (AusAID), Government of the People's Republic of Bangladesh, Canadian International Development Agency (CIDA), Embassy of the Kingdom of The Netherlands (EKN), Swedish International Development Cooperative Agency (Sida), and the Department for International development (DFID), UK. The authors gratefully acknowledge these donors for their support and commitment to ICDDR,B's research effort.

The authors also express gratitude to the concerned upazila health and family planning officers who extended their full cooperation by providing with data. The assistance from other hospital staff of the concerned health complexes is duly acknowledged. Finally, the authors are indebted to those who reviewed the paper.
